# Co-production with marginalised workers: working with homecare workers and managers caring for people approaching end-of-life

**DOI:** 10.1186/s40900-025-00814-z

**Published:** 2025-12-06

**Authors:** Zana Bayley, Cat Forward, Helene Elliott-Button, Justine Krygier, Caroline White, Mark Pearson, Liz Walker, Colin Moss, Jamilla Hussain, Paul Taylor, Jane Wray, Helen Roberts, Miriam J. Johnson

**Affiliations:** 1 Faculty of Health Sciences, The University of Hull, Hull, HU6 7RX UK; 2 Health and Social Care Workforce Research Unit, King’s College London, London, UK; 3 Wolfson Palliative Care Research Centre, Hull York Medical School, The University of Hull, Hull, UK; 4 Involve Hull, Institute for Clinical and Applied Health Research, The University of Hull, Hull, UK; 5 Bradford Teaching Hospitals Foundation Trust, Bradford, UK; 6 St Luke’s Hospice, Sheffield, UK; 7 Sheffield Centre for Health and Related Research, University of Sheffield, Sheffield, UK

**Keywords:** Palliative care, Training, Co-design, Co-creation, Co-production, Homecare

## Abstract

**Background:**

Co-production is important due to its effectiveness in creating relevant and meaningful outputs for use in social and healthcare practice, however, frontline staff such as homecare workers (also known as aides, personal assistants or domiciliary care workers providing paid care within the home) are a key group within the social care workforce who are under-represented in this approach. Here, we report our coproduction process engaging with this workforce to develop training resources for workers providing end-of-life homecare.

**Aim:**

To co-produce training resources with homecare workers and their managers to support and educate workers delivering end-of-life homecare using evidence from our larger qualitative interview study.

**Methods:**

We conducted a series of 12 co-production workshops with UK-based homecare workers and managers (partners) to design training resources and recommendations for homecare providers informed by research findings. We adopted the five key principles of co-production: Sharing of power; Including all perspectives and skills; Respecting and valuing knowledge; Reciprocity; and Building and maintaining relationships. A co-production advisory group of homecare workers as well as the workshop partners gave valuable oversight throughout the workshop series.

**Results:**

77 partners (31 homecare workers, 46 managers) participated in 12 workshops (one face-to-face; 11 online). Our approach enabled power-sharing, inclusivity, respect, collaboration and reciprocity, relationship-building, and identification of effective flexible approaches to co-production. Specific forms of training resources were co-created. Training recommendations (content, delivery formats, access during working hours, etc.) were also developed together. Challenges were non-attendance and lack of engagement by some partners during sessions.

**Conclusion:**

These workshops are the first, to our knowledge, to successfully co-produce end-of-life care training resources with homecare workers and managers, a poorly represented workforce in co-production. Challenges included inconsistent attendance and poor engagement by a minority of partners. The five key principles of co-production enabled true engagement with the process, thereby enriching the final outputs.

## Abstract (plain language summary)

**Background:**

Access to personalised homecare is crucial for people wishing to remain in their own homes when approaching end-of-life. Most home-based care within the UK is provided by paid, unqualified (a standard professional qualification is not required) carers, often called homecare workers or domiciliary carers. End-of-life care addresses social, psychological, emotional, and physical needs but there is little training that is accessible or available to this workforce. In addition, this workforce is rarely, if ever, involved in the co-production of training resources.

**Methods:**

We used an approach called co-production to partner with homecare workers and their managers to co-create end-of-life care training resources. The parent study for these workshops included interviews with care recipients and their families, health professionals, and homecare workers and managers and evidenced the key areas of training needed for homecare workers when delivering this type of care. We used this knowledge to co-produce training resources and a set of recommendations for training.

**Results:**

Several key aspects of this co-production approach were effective in engaging a workforce rarely considered in co-production activities. There were some challenges such as lack of engagement from some partners.

**Conclusions:**

As far as we know, this is the first example of successful co-production work partnered with homecare workers and managers to co-create meaningful training material for use in the workplace. Our experience may be helpful for others wishing to conduct co-production work with rarely involved partners.

## Background

In the UK, homecare workers, (also called aides, care assistants, domiciliary carers, personal assistants or other titles in the UK and overseas), are a crucial workforce providing essential individualised, and relational basic personal care and support to people in their own homes [1, 2]. As people approach the end-of-life, many need care and support from homecare workers, particularly those wishing to remain (and die) in their own home.

Training is crucial to enable homecare workers to work safely, confidently, and effectively, and can improve care quality, staff retention, and overall service user satisfaction [3]. Providing quality end-of-life homecare and managing the challenging emotional and psychological impact of such work requires staff training informed by real-world examples and experiences [4, 5]. In the UK, end-of-life care training for the homecare workforce varies in terms of availability and quality [1] with no standardisation for workers and providers. However, ongoing, end-of-life care training is needed to help improve provision of this care [6] for example, in managing symptoms like breathlessness or pain [7].

Co-production is an emerging field in health and social care [8, 9], including the co-creation of educational materials [10–12]. In this paper we adopt the definition of co-production as the process of co-developing a solution to a problem [13]. Co-production is more effective, relevant, engaging, and impactful, and outputs more likely to be accepted and used in health and social care [27, 28]. Despite the increased interest in this approach, there are few reports about the involvement of care services in co-developing solutions, reducing the ability for others to learn from best practice and prior experience [14], with few practical illustrations of co-creation approaches [8]. To our knowledge, none have included homecare workers. This workforce is often overlooked in health and social care service research and its implementation [15]. This is despite over 14,000 recognised homecare organisations providing care to over one million people in the UK [16] and homecare provision becoming a key issue worldwide due to an ageing population, increased family mobility, and increasing complexity of care required within the home setting [17, 18]. Knowledge mobilisation describes the generation, sharing and use of evidence within health and social care [19]. Here, we report how we designed and delivered successful co-production workshops with homecare workers and managers as partners to share and use evidence to develop focused, relevant, and appropriate training resources.

### Aim

To describe the process of co-producing training resources with homecare workers and their managers to support and educate workers in delivering end-of-life care.

### Parent study background

The SUPPORTED study [20], explored the experiences and training needs of homecare workers providing homecare at end-of-life and identified the topic areas and delivery approaches for training. Detailed methods and findings of this study are reported elsewhere [21–23]. In summary, we found that homecare workers are not routinely trained or knowledgeable about caring for clients approaching end-of-life, and have little engagement or involvement with any other professionals providing care, such as community nursing, hospices, therapy services or local charity support. The topic areas for training content and delivery considerations are shown in Table 1 below.

**Table 1:** Identified training topics and training resource formats

Agreed Topic Areas for Training	First steps into end-of-life care ‘Just a care worker’ – understanding what you bring Practicalities of delivering care at end-of-life Looking after yourself Homecare worker as a professional End-of-life care and the unexpected The final months of life – what might it look like? Different conditions – what you might see Not just the physical – psycho-social and spiritual care Communication skills Working with those important to the people you care for Working with other professionals Effective management – beyond the team Interacting as a team Advanced communication skills Expanding the role of the homecare worker
Delivery Considerations – Agreed Formats	Slide decks for face-face teaching Narrated slide decks for remote learning What if…? Cards for adhoc ‘bitesize’ learning and supporting supervisions, debriefs, group and individual learning

### Co-production methods

Co-production uses a participatory approach, where project facilitators and partners-with-experience work collaboratively, sharing power and valuing each other’s different expertise [24]. We adopted five key principles of co-production: Sharing of power; Including all perspectives and skills; Respecting and valuing knowledge; Reciprocity; and Building and maintaining relationships [25].

#### Recruitment

We invited homecare workers and homecare agency managers across England from organisations who had engaged in the SUPPORTED study, as well as using social media and established contacts known to the project team to identify other partners. Skills for Care, a national workforce development organisation, also promoted the workshops through their networks. As this population are under-represented in service development or research, getting access to, and positive responses from homecare providers was difficult. They were unfamiliar with the structure and purpose of coproduction, which, together with a varied and irregular work pattern, for example, no regularly time-tabled shifts, and workers on zero hours contracts, meant that sustained effort throughout the 7-month period was required to gain and maintain sufficient numbers.

From 133 people who registered to participate in the workshops, 31 homecare workers and 46 agency managers took part in 12 workshops (one face-face; 11 online) in four rounds of three parallel workshop sessions over a period of seven months during 2024 to enable the co-production of training resources. 37 people attended more than one session but attendance was unpredictable (see Table 2).

**Table 2:** Workshop attendances

Workshop round	Agreed to attend	Actual attendance	Attendance for each workshop	Previously attended
1	30	19	10, 6, 3	-
2	32	21	3, 7, 11	5
3	42	20	6, 7, 7	16
4	39	17	5, 7, 5	16

**Fig. 1:**
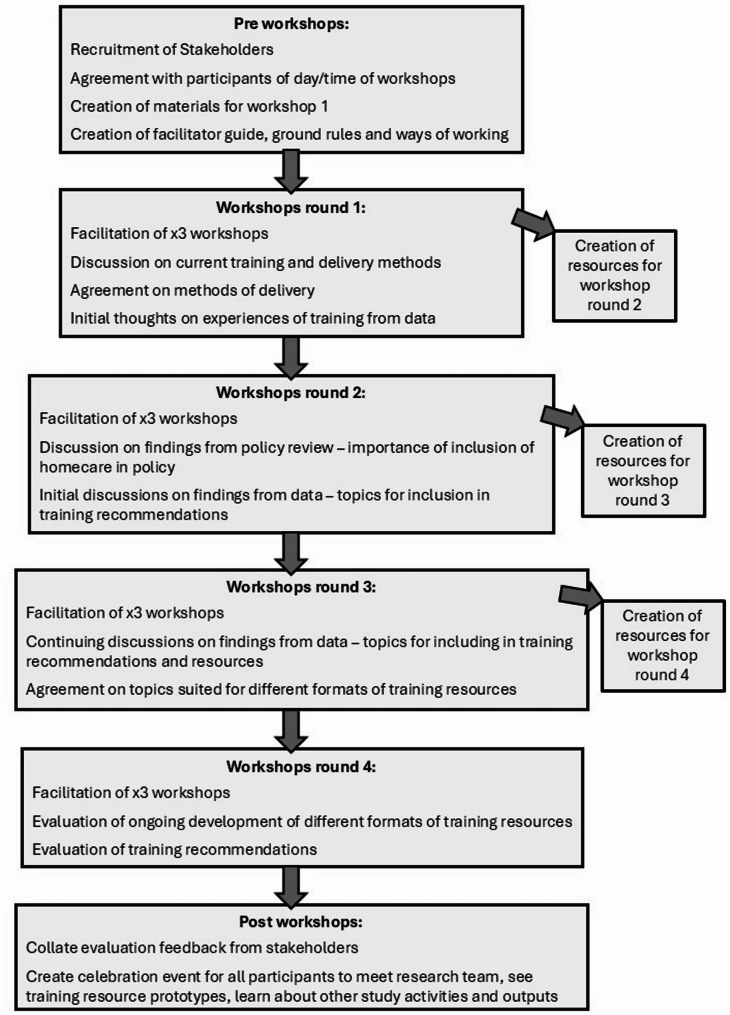
Workshop stages

Workshops were chosen to allow space for discussion, reflection, and refinement [26]. Planning of the co-production workshops was supported by a service user advisory group comprising of homecare workers and carers (family or friends) of people who had received care. They provided important feedback such as the use of parallel sessions to ensure managers and homecare workers were given separate spaces to openly contribute. The stages of the workshops are illustrated below in Fig. 1.

Our project team members included five experienced qualitative researchers with professional backgrounds in education, occupational therapy, social work, medicine, nursing, and social care, as well as transferable skills in facilitating workshop discussions and dialogue. One, who had professional training and experience in education, took responsibility for creating workshop resources and facilitator guides. A facilitator’s guide was produced for every workshop to ensure consistency. These detailed each activity including suggestions for stimulating discussion. Two team members facilitated each workshop to ensure partners felt included, and their perspectives were valued, rather than just perceived as an add-on to enhance research quality [29]. Having two facilitators reduced the risk of missing any important information or insight shared by partners, as a form of member-checking the information shared.

### Data collection

Each workshop was video recorded in addition to facilitators making notes to aid accurate recall and summary of key points and decisions made. These were then used to help produce initial versions of the resources, and plan for the subsequent rounds of workshops where resources were refined.

## Results

The workshop series enabled us to report three key areas. Firstly, the impact of our techniques on the co-production process, secondly the impact of co-production on the training resource outputs, and thirdly, the impact of participation in the co-production activities on the partners.

### Impact of co-production techniques

The use of the various techniques helped foster knowledge sharing and collaborative working to co-produce training resources. The techniques we used which evidence each of the co-production principles [19] are shown in Table 3.

**Table 3:** Summary of co-production principles and techniques used

Co-production principles	Techniques used
*Sharing of power - The research is jointly owned*,* and people work together to achieve a joint understanding*	Researcher role designated as facilitator Partners involved in the design and content of workshop series Sharing of decision-making relating to resource production
*Including all perspectives and skills - Make sure the research team includes all those who can contribute*	Facilitators supported with guidance on how to manage group sessions Accessible workshop design through format and materials used Facilitators’ knowledge and skills in active listening and probing to encourage contributions Implementing ‘Ways of Working’ plan to facilitate inclusion Use of technology (i.e. Microsoft Teams) to enable partners to contribute
*Respecting and valuing the knowledge of all those working together on the research - Everyone is of equal importance*	Facilitators encouraged group discussions Workshop materials provided in understandable language and style in advance Direct acknowledgement of expertise and experiences of partners Recording of session and note-taking to enable use of knowledge shared Partners received payment for their contribution
*Reciprocity - Everybody benefits from working together*	Workshops achieved consensus among partners re: training materials design and content Partners co-produced a list of recommendations for training Partners collaboratively decided on importance of training areas Partners gained knowledge on purpose of research, research methods, and co-production techniques
*Building and maintaining relationships*	Accommodation of partners’ needs in relation to scheduling of workshops, materials provided, structure of sessions Creation of inclusive and respectful workshop environment Regular communication throughout workshop series on impact of involvement and processes of co-production Invitation to end of research celebration event

We dedicated preparatory time to develop materials for supporting the workshops and guiding partners; a key component of effective co-production [12]. We observed that the ‘Ways of Working’ and ‘Ground Rules’ materials helped us to establish a shared understanding of the workshop environment, especially important as many of the partners had not engaged in co-production activities before. The materials covered issues such as confidentiality and respect, encouraged inclusion, active listening, use of cameras and microphones for online sessions, and recording of sessions. We reminded partners of these ways of working, which helped us facilitate the workshops in a friendly, open, and inclusive way, and develop a rapport within groups. The partners advised us that they appreciated a structure with facilitators to guide discussion and to encourage people to contribute. These ground rules allowed us to create a safe and supportive space for us to monitor group dynamics, ensuring all were given time and space to share their perspectives [30].

Resource packs were created for each session, where we presented the information from the SUPPORTED study in accessible formats; these were shared in advance of each workshop. This was planned to help partners feel more prepared and confident for the session’. Partners also told us that it helped them to acquire new knowledge and understanding around the SUPPORTED study’s findings. They felt better able to engage and contribute to the workshops, resulting in higher quality co-produced outputs. We dedicated some of the early workshop sessions to providing an insight into the previous research process from the SUPPORTED study, explaining terminology and practices such as data analysis. This was a form of knowledge mobilisation and making the process more relevant, which has been argued to be an effective strategy for co-production in health research [17].

Partners were paid for their time in line with the National Institute of Health Research (NIHR) and best practice for co-production guidelines. Payment encouraged recruitment and participation as homecare workers advised that they appreciated this, particularly as we also paid for time to read and review the material prior to the workshops. Payments also helped to demonstrate that we valued their time and expertise and provided a tangible benefit for their involvement; particularly relevant for a low-paid workforce.

We used our series of workshops to enable a larger number of perspectives to be heard across the groups, and to develop ideas as the resources were developed. The workshops were arranged at mutually convenient times. Some workshops were held at the weekend or during the evening to accommodate the varying needs of a workforce who provide a 24/7 service. Our approach of having multiple workshops, each building on the previous session, enabled us work at a slower and focused pace [31] and develop relationships, as we found over half of partners attended more than one workshop.

### Impact of co-production

The various strategies we employed to build rapport, support inclusion, and work in a mutually beneficial workspace enabled significant homecare worker input to the development of the training resources throughout the workshops.

The first round of workshops was focused on initial ideas and concepts for the resources. The proposal to ensure some training was targeted at managers arose from concerns that as often the gatekeepers for staff to undertake training, they need to have the knowledge and skills to do so. The partners also helped to shape the length, format, and level of training – advising that training should be tiered to suit different stages of career, be available in different formats depending on whether it is face-face or remote training, and different lengths to suit a “coffee break” snippet or longer dedicated training sessions.

In the second round we shared some initial findings from the research study, which prompted discussions on the key topics that training should cover, influencing our curriculum document. The partners also worked with us on our policy review findings and agreed that policy around end-of-life care must include community-based care workers, because of the crucial work they provide for people who often want to be at home when approaching end-of-life.

We presented more findings from the SUPPORTED study for the third round of workshops. This enabled partners to consider specific areas of training that we could develop, for example, managing conflict, emotional burden, and signs of dying. We were also able to work together to understand which training was better suited to different formats, for example, partners were keen that any training focused on communication such as working with family members, should be designed for a face-face delivery style i.e. PowerPoint slides for a manager/trainer.

In the final round of workshops, partners were able to advise on draft versions of training material we had produced following their suggestions in round 3. This critique enabled us to modify and refine our resources. We were also able to share a draft version of our training recommendations document, which we were able to collectively improve to be more reflective of what the research and our partners feel is most useful and effective.

The culmination of all the workshops enabled us to generate a suite of resources including a training recommendations booklet, Powerpoint slide decks, PowerPoint videos with voiceover, and a series of printable postcards entitled “*What if…”* cards which detail a response to a fictional question based on the research data and the workshops discussions e.g. “*What should I do if my client says something inappropriate to me?*”.

We recorded the impact of the partner’s recommendations within each round of workshops separately and have summarised these in Table 4.

**Table 4:** Impact of Co-production on outputs

Partner Recommendation	Impact
**Workshop 1**	
Training managers is crucial to better support homecare workers, and to potentially provide in-house end-of-life care training	Separate recommendations for end-of-life care training have been created for managers
End-of-life care training should be at distinct levels as new homecare workers may be overwhelmed by too much content on end-of-life care	Adaptation of training recommendations into three levels of ‘first weeks’, ‘first months’ and ‘advanced’ for homecare staff, including training aimed at managerial level to support workers when learning about end-of-life care
Any materials should be easily accessible	We will ensure all end-of-life care training materials are freely accessible online and agencies can print off resources to give to staff who cannot access online
Training sessions should be different lengths of time to allow for remote learning and in-person training	Presentation length was reduced, with space for lengthening or merging materials to create longer sessions by agencies if required to meet specific end-of-life care training needs locally.
Any materials need to include case studies, scenarios, and problem-based learning	Case studies from our data, input from expert writers knowledgeable of end-of-life care, and fictional situations have been included in the training
Material should be permanently available, for refresher training even after completion	Material will be freely available at any time online
Training should be personalised to suit learners –delivery, content, approach, assessment	Material can be modified by managers to suit their individual learners and context of their own location and staff
Consider accreditation of any training content	This is beyond the scope of this project, but it has been included as part of our policy recommendations
Ensure communication is a key component of any end-of-life care training	Training in communication has been included within the end-of-life care training resources
**Workshop 2**	
Identified reasons for homecare workers to be included in policy on community-based end-of-life care	Some of the points raised have been integrated into our policy recommendations
Identification and agreement on critical areas of training needs for homecare workers, based on findings from research data themes	Used in first draft of training recommendations and considered when creating list of training resource topics
**Workshop 3**	
Further dentification of important training areas through discussion and activities drawing on themes from interview data analysis findings	Used to generate list of end-of-life care training resources to be created, and development of the training recommendations document
**Workshop 4**	
Critiqued draft recommendations with guidance on improvements	Critique used to modify recommendations for training curriculum
Critiqued draft formats of training resources with guidance on improvements	Resources modified according to workshop suggestions including colour, layout, format. Recommendations to keep text levels minimal, employing colourful and fun design, include interaction, space for discussion and reflection, and quotes where possible shared with resource writers. Allowance for mangers to modify where appropriate. Recommendations influenced final designs including hearing the authentic voice of experts including homecare workers and managers.

## Discussion

We report a successful collaboration which valued each other’s expertise and knowledge. Co-production is a flexible and holistic method of developing educational materials [32] more likely to be adopted and usable [12], but which comes with its own challenges and tensions [33]. We managed challenges and tensions, being mindful of the difficulties of working with a workforce poorly represented in co-production, who benefitted from adapted strategies for inclusion within co-production [34]. We had to build trust within the homecare workforce, aware that the lack of experience and knowledge of co-production could cause mistrust and reluctance to contribute [27].

Changes to practices and cultures are needed to enable the application of co-production principles [33]. The specific support needs required for collaborations with under-represented groups such as the homecare workforce [15] need to be understood, and a bespoke plan made, which is flexible around the context, content, and the cohorts engaged [34].

Co-production has enabled previously marginalised service users to become equal partners [35]. This was true in our experience with the homecare workforce. By incorporating co-production principles into the planning and delivery of the workshops we evidence tangible outcomes that were directly influenced by homecare workers and homecare managers. These outcomes were not just in terms of the training resources produced, but also in the mutual benefits for both the project team members and the homecare workers and managers involved, who were able to develop a closer relationship and understanding about their different perspectives and knowledge base.

We aimed to co-produce training resources with homecare workers and homecare managers, as we wanted our resources to be grounded in their experiences and expertise and be useful within the care sector. To our knowledge, this is the first study to develop training resources for homecare workers that has not only used data directly sourced from homecare workers and those associated with home care at end-of-life but also partnered with homecare workers and managers to collaboratively co-produce resources.

This report provides an example of a successful method used to create training resources for health and social care service providers who are often not included in collaborative and inclusive practice, have no regulation regarding what end-of-life training (if any) they should receive, and yet are arguably one of the most essential care providers of end-of-life homecare. Previous studies around education and training for end-of-life care have not been developed with this workforce in mind, and many are not able to access training due to costs, availability, or lack of support by their employers. Our study has endeavoured to address that omission to benefit this crucial and yet often unheard workforce.

### Challenges and lessons learned

We faced challenges and learned lessons about co-production with an unfamiliar and marginalised group. As the workshops were offered nationally, we could not offer in-person options in every round. This may have been a barrier for some who feel less confident or lack the necessary technology or digital confidence to contribute online. However, online sessions, and acknowledging that homecare can be a 24/7 job, often alongside other caring and family commitments, enabled opportunities for participation at various times of the day and week with sessions agreed collaboratively with partners. Also, we were able to offer this opportunity to people nationally, allowing us to include experiences from different regions, which may commission homecare services at end-of-life differently.

The sustained commitment of our partners is a key strength, as most opted to return for subsequent workshops, which was not a requirement or anticipated by the team. Having stages of workshops enabled those who returned to see the generation and growth of the training resources, provided confirmation that their contribution materially affected the development of resources, and enabled a greater appreciation of the value and purpose of co-production.

Due to the study timeframe, the resources could not be made available for a fuller appraisal before the end of the workshops, however all attendees will be able to access these once completed. This was a limitation of the study design itself, where the timing of the workshops should have coincided better with the physical production of the resources to allow more testing of content, design, and accessibility.

Reluctance to contribute, and non-attendance need to be considered when designing co-production activities with a workforce who are time-poor, engaged in an unpredictable work environment, and who have little experience of such involvement which might cause anxiety prior to attending, or distrust in the genuineness of the process (fear of tokenism). We needed to recruit homecare workers and managers throughout the period to ensure we had enough for each round. Possible strategies to mitigate these difficulties in the future may be to provide more guidance and support on what to expect within a co-production workshop setting, and background on co-production as a method for applying new knowledge. This would help partners to be more confident in their expectations of what was required in the workshops, and the benefits of their engagement. Also, the format of a group discussion online is not necessarily the preferred format for some people, and 1–1 sessions with facilitators could be considered, depending on numbers and practical issues around workload.

We were not able to robustly evaluate the process with our partners which limited our understandings of their perspectives on the workshop format, process, and outcomes. We did not conduct interviews exploring their experience of the process, and although we did conduct a short survey, so few people responded (11/40), this gave little useful information. Low completion of the evaluation survey may reflect their marginalised status, as people from minority groups, low-literacy, and/or language and access barriers have been associated with poor responses in surveys [36]. There may be other reasons for non-response, including unfamiliarity of workshops and evaluation processes; concern that negative feedback would not be acted upon or valued; lack of priority given by a time-poor workforce; and difficulties in quantifying views with a Likert scale or expressing them in written form in the free text [37, 38]. Lower education levels and low incomes and non-response have been previously linked [39, 40]. The low response from the survey underlines the need for a more direct engagement with people less used to being asked for feedback, such as interviews, or individual contact to assist survey completion.

## Conclusion

We provide an example of co-production of training resources with an under-represented social care workforce, evidencing adopted strategies that enabled effective engagement over a sustained period. We explain how we engaged with these partners adopting the five key principles of co-production, enabling collaboration in the creation of training resources to support homecare workers delivering care at end-of-life. We share the learning points and challenges which may help others planning similar co-production activities with under-represented groups. Finally, we acknowledge the need to develop current national strategies for increasing social care service improvement and research engagement with consideration of the uniqueness of the homecare workforce [15].

## Data Availability

The workshop phase of the study did not require ethics approval, and there are no data available from the workshop sessions. The training resources will be freely available online, when completed. The data from the larger study are available on request by authorised researchers following completion of a data sharing agreement. To request access, contact the study authors, or [worktribe@hull.ac.uk] citing Worktribe Output ID 5179695.
